# Liraglutide-associated depression in a patient with type 2 diabetes: A case report and discussion

**DOI:** 10.1097/MD.0000000000037928

**Published:** 2024-05-03

**Authors:** Yangliuqing He, Fenrong Liang, Yiming Wang, Yuhan Wei, Tianpei Ma

**Affiliations:** a Clinical Medicine College of Guizhou Medical University, Guiyang, China; b Department of Psychiatry, Affiliated Hospital of Guizhou Medical University, Guiyang, China.

**Keywords:** case report_4_, depression_1_, GLP-1 receptor agonists (GLP-1RA)_3_, liraglutide_2_

## Abstract

**Background:**

Glucagon-like peptide-1 receptor agonists (GLP-1RAs) like liraglutide are primarily used for managing blood sugar levels in type 2 diabetes and aiding weight loss. Typically, their adverse effects are gastrointestinal, with limited exploration into their impact on mental health.

**Case presentation:**

This report examines a 39-year-old male with type 2 diabetes who developed depressive symptoms after starting liraglutide for glycemic control and weight reduction. Symptoms included poor mood, irritability, decreased interest and energy, progressing to sadness, low self-esteem, and physical discomfort. A clinical diagnosis of a depressive episode was made, coinciding with the initiation of liraglutide.

**Intervention and outcome:**

The patient depressive symptoms significantly improved within a week after discontinuing liraglutide and starting antidepressant therapy. This suggests a possible link between liraglutide and depression, despite considering other factors like diabetes-related stress.

**Discussion:**

The report explores potential mechanisms, such as GLP-1RA effects on glucose fluctuations and dopamine modulation, which might contribute to depressive symptoms. The influence on the brain reward system and the reduction in cravings for addictive substances after GLP-1RA use is also discussed as a factor in mood regulation.

**Conclusion:**

This case highlights the necessity of being vigilant about potential psychiatric side effects, particularly depression, associated with GLP-1RAs. The rarity of such reports calls for more research to investigate and understand these implications further.

## 1. Introduction

Glucagon-like peptide-1 receptor agonists (GLP-1RAs), through specific activation of the GLP-1 receptor, enhance insulin secretion in a glucose (GLU)-concentration-dependent manner while effectively suppressing the release of glucagon. This dual mechanism contributes to the reduction of blood GLU levels. Additionally, higher doses of GLP-1RAs can reduce food intake and increase satiety by delaying gastric emptying and suppressing the appetite center in the central nervous system.^[1]^ Liraglutide, as a GLP-1RA, has been approved by the FDA (Food and Drug Administration) for the control of blood sugar in patients with type 2 diabetes and for weight loss in obese patients.

In the clinical application of Liraglutide, adverse events are mostly reported in the gastrointestinal system, with nausea and diarrhea being the most common. Vomiting, constipation, abdominal pain, and dyspepsia also occur with relative frequency. Additionally, headaches, nasopharyngeal discomfort, and hypoglycemia^[2]^ are common adverse reactions. Few reports have associated the use of Liraglutide with gallstones, acute pancreatitis,^[3]^ and acute renal injury.^[4]^ There were reports of increased suicidal ideation with the use of Liraglutide; however, a recent systematic review by the European Medicines Agency stated that there is no causal relationship between GLP-1RAs and suicide.^[5]^ Further more, studies suggest potential antidepressant effects of GLP-1RAs,^[6–8]^ which may be related to their involvement in the regulation of inflammation^[9,10]^ and hormones such as cortisol and thyroid hormones.^[11]^ Here, we report a case of a young male who developed depressive symptoms after using Liraglutide for blood sugar control and weight reduction. This patient, a young male with no history of depression, experienced worsening depressive symptoms after increasing the dose of Liraglutide. His symptoms improved markedly after discontinuing Liraglutide and starting antidepressant medication. To our knowledge, there are currently no reports on Liraglutide causing depression. Therefore, our case report will fill this gap, providing preliminary evidence of a potential link between Liraglutide and depression.

## 2. Clinical data

### 2.1. Basic medical history

A 39-year-old male patient, with a graduate degree and employed as a company staff member, was admitted to our hospital on December 4, 2023, due to “poor mood for over 2 months.” Two months prior to admission, the patient was diagnosed with type 2 diabetes at another hospital and was prescribed Liraglutide 0.6 mg subcutaneous injection once daily (qd) and Dapagliflozin 10 mg qd for blood sugar control and weight loss. Subsequently, he began to experience symptoms of mood deterioration, primarily characterized by inexplicable sadness, irritability, loss of interest, and decreased energy. Two weeks later, due to insignificant weight loss, the dose of Liraglutide was increased to 1.8 mg qd during a follow-up visit at the same hospital. This led to significant weight loss, accompanied by increased fatigue, feelings of sadness, inferiority, and uselessness, worry about his illness, nausea, diarrhea, palpitations, chest tightness, and trembling hands. The patient suffered from poor sleep quality, difficulty in falling asleep, light sleep, early waking, and inability to fall back asleep, resulting in non-restorative sleep. Under the treatment with Liraglutide and Dapagliflozin, the patient lost approximately 20 kg over 2 months. One week after discontinuing Liraglutide on his own, his symptoms of nausea and diarrhea completely resolved, but other symptoms showed no significant improvement. He then visited our outpatient clinic and was admitted for a “depressive episode.” In terms of past medical history, the patient was diagnosed with hypertension (details unspecified) over 2 years ago and was regularly taking Metoprolol Succinate 47.5 mg qd, Amlodipine Besylate 5 mg qd, and Irbesartan 150 mg qd for blood pressure control, which he reported as effective. He was diagnosed with dyslipidemia (details unspecified) over 2 years ago and intermittently took Atorvastatin calcium 20 mg every night for lipid-lowering, with unknown lipid control status. Two months prior, he was diagnosed with type 2 diabetes (details unspecified) and was prescribed Liraglutide 1.8 mg subcutaneous injection qd and Dapagliflozin 10 mg qd for blood sugar control. He discontinued Liraglutide a week before on his own, reporting acceptable blood sugar control. No other notable history was reported. Regarding personal history, the patient is an only child, had a good development since childhood, and works in a travel company. He got married at the age of 26, has a good relationship with his spouse, and has 1 healthy daughter. He occasionally drinks alcohol and has a 20-year history of smoking about 20 cigarettes per day. Before the illness, the patient was outgoing and had good interpersonal relationships, many friends, and strong work abilities. In terms of family history, he denies any history of mental disorders in 3 generations of his family.

### 2.2. Physical examination

Upon admission, the patient temperature was 36.3°C, heart rate 104 beats/min, respiratory rate 20 breaths/min, and blood pressure 135/91 mm Hg. Examination of the skin, mucous membranes, heart, lungs, and abdomen did not reveal any significant abnormalities. Neurological examination showed no positive signs. The mental status examination revealed that the patient was alert with intact orientation but appeared slightly disheveled. He exhibited a distressed expression and sighed frequently, breaking down in tears when discussing adverse reactions to Liraglutide for diabetes treatment. He was passively cooperative, answered questions relevantly, but showed slow response, poor concentration, slow speech, reduced speech volume, and a low tone. His emotional responses were congruent, with a predominant mood of sadness, reduced interest, lack of motivation, and decreased self-esteem, along with irritability. There were no delusions, hallucinations, or perceptual disturbances. His thought process was coherent, with ideas of reference, believing that colleagues, family, and friends thought he was “no longer useful.” He reported feeling mentally sluggish, slow in action, decreased energy, easily fatigued, and unable to concentrate at work. The patient displayed negative speech and behavior, suffering from insomnia characterized by difficulty in falling asleep and early waking. His intelligence appeared normal, and he had insight into his condition.

### 2.3. Laboratory and auxiliary examinations

Liver function + Kidney function + Electrolytes: TP 48.6 g/L, albumin 32.2 g/L, globulin 16.4 g/L, creatinine 54 µmol/L, uric acid 440 µmol/L, GLU 7.95 mmol/L, cystatin C 1.14 mg/L, K 3.22 mmol/L, calcium 2.03 mmol/L, bicarbonate 20.7 mmol/L, alanine aminotransferase 43.2 U/L, aspartate aminotransferase 20.3 U/L. Lipid profile: triglycerides 9.16 mmol/L, total cholesterol 6.89 mmol/L, high-density lipoprotein cholesterol 0.84 mmol/L, low-density lipoprotein cholesterol 3.42 mmol/L. Glycated hemoglobin was 9.72%. Routine blood and biochemical tests revealed no significant abnormalities. Near-infrared brain function imaging suggested the possibility of a depressive state, and further clinical examination was recommended. Psychological assessment: Hamilton Depression Scale score of 30, indicating moderate depression; Hamilton Anxiety Scale score of 26, indicating significant anxiety.

### 2.4. Treatment course

Regarding treatment, the patient continued his previous blood pressure and blood sugar control regimen for diabetes and hypertension. Due to poor control of blood sugar and lipids, he was advised to add Metformin extended-release tablets 1 g twice daily and Fenofibrate capsules 0.2 g qd for symptomatic treatment following an endocrinology consultation. The patient initial low potassium was considered related to reduced food intake, and after encouragement to eat more and potassium chloride supplementation, his blood potassium levels normalized. In terms of psychiatric treatment, he was initially prescribed Escitalopram Oxalate 10 mg qd and Alprazolam 0.4 mg every night to improve mood and sleep. After 1 week of medication, the patient reported an improvement in mood. To further improve symptoms, the dose of Escitalopram Oxalate was increased to 20 mg qd. On the day of discharge, the patient reported significant improvement in mood, feeling calm and relaxed, regaining control over his life, resuming outdoor activities, good appetite, and better sleep quality. At discharge, the patient Hamilton Depression Scale score was 6, indicating no depressive symptoms, and Hamilton Anxiety Scale score was 5, indicating no anxiety symptoms. A 2-week follow-up showed that the patient mood remained stable.

As shown in Table 1, we present the detailed clinical course and treatment outcomes of a patient following Liraglutide administration.

**Table 1:** Clinical course and treatment outcomes in a patient with liraglutide-associated depression.

Time frame	Event/milestone
Over 2 yr prior to admission	Diagnosed with hypertension and dyslipidemia, managed with medications
Over 2 mo prior to admission	Diagnosed with type 2 diabetes; started on Liraglutide 0.6 mg qd and Dapagliflozin 10 mg qd
2 wk after diabetes treatment	Symptoms of poor mood began, with gradual worsening
After initial treatment period	Increased Liraglutide to 1.8 mg qd; significant weight loss, fatigue, and worsening depression
1 wk before admission	Discontinued Liraglutide; nausea and diarrhea resolved, other symptoms persisted
Admission	Admitted to the hospital with “depressive episode”
	On admission: Physical and neurological exam largely normal; signs of depression and anxiety present
	Laboratory tests: Showed various results including elevated blood sugar and lipid levels
	Psychological assessment: HAMD score of 30 (moderate depression), HAMA score of 26 (significant anxiety)
During hospital stay	Continued management for diabetes and hypertension; prescribed Escitalopram Oxalate 10 mg qd and Alprazolam 0.4 mg qn for mood and sleep
1 wk after treatment adjustment	Improved mood and sleep; to further improve symptoms, the dose of Escitalopram Oxalate was increased to 20 mg qd
On discharge	Significant improvement in mood; HAMD score of 6, HAMA score of 5
2 wk post-discharge	Stable emotional state maintained

HAMA = Hamilton Anxiety Scale; HAMD = Hamilton Depression Scale; qd = once daily; qn = every night.

## 3. Discussion

In this case, factors other than Liraglutide, such as diabetes itself, stress related to the diagnosis and prognosis of the disease, psychological events, and somatic discomfort following Liraglutide use, may also have contributed to the onset. However, the patient exhibited depressive mood before the onset of physical discomfort and did not show significant mood improvement when the physical symptoms eased. Furthermore, these factors do not correspond to the aggravation of the patient depressive mood and the significant improvement shortly after discontinuing Liraglutide. Indeed, Escitalopram Oxalate treatment can help improve mood, but clinical practice suggests a 2 to 4 weeks delay in the onset of SSRIs, whereas the patient showed significant mood improvement within a week of medication, suggesting a close association with the discontinuation of Liraglutide.

Depression associated with GLP-1RA use is rarely reported, and its relationship with depressive symptoms remains unclear. Regarding this young male depression, we speculate it might be related to: Blood sugar instability caused by GLP-1RA; Reduced dopamine levels due to GLP-1RA modulating dopamine activity and reuptake, leading to anhedonia.

Studies suggest that fluctuations in blood GLU levels can affect mood and increase the risk of depressive symptoms.^[12,13]^ GLP-1RAs, while controlling blood sugar by increasing insulin secretion and reducing glucagon production, can also cause GLU fluctuations,^[14]^ with hypoglycemia being a common adverse reaction in clinical use.

Additionally, GLP-1RAs can reach multiple brain areas, directly binding to different GLP-1 subtypes of receptors located in the brainstem, lateral septal nucleus, and hypothalamus, participating in the regulation of neural pathways related to feeding, reward, and energy expenditure.^[15]^ Notably, GLP-1RAs stimulate receptors in the ventral tegmental area, increasing the activity of dopaminergic neurons and the expression of dopamine transporters in the brain. Increased expression of dopamine transporters, responsible for the reuptake of dopamine into neurons, may result in reduced free dopamine levels in certain brain areas (like the lateral septal nucleus and striatum).^[16]^ Dopamine plays a vital role in the brain reward system, which is associated with motivation, mood, and craving for food. Its dysregulation may lead to anhedonia and other depressive symptoms.^[17]^ In fact, studies have shown reduced cravings for addictive substances after using GLP-1RAs,^[18]^ possibly related to their effect on the dopamine reward system.^[19]^

In summary, this case report highlights a young male with Liraglutide-associated depression. Given the scarce reports of GLP-1RA-related depression and the unclear mechanism between GLP-1RA use and depression, further case reports and laboratory studies are required to enhance understanding of GLP-1RAs and prevent adverse reactions, possibly identifying new targets for the treatment of depression. However, until such findings are more established, it is crucial to monitor emotional changes in patients using GLP-1RAs to adjust treatment plans promptly before the condition worsens.

## 4. Conclusion

This case highlights the complexity of managing diabetes with GLP-1RAs, particularly in patients who may be susceptible to mood disorders. The patient improvement upon discontinuation of Liraglutide and initiation of antidepressant therapy underscores the need for healthcare professionals to be vigilant about the psychological well-being of patients undergoing GLP-1RA therapy. Moreover, it raises questions about the neurochemical effects of GLP-1RAs, particularly regarding their influence on the dopamine system and its potential implications for mood regulation.

While the therapeutic benefits of GLP-1RAs in managing diabetes and obesity are well-established, this case suggests a need for a more holistic approach that considers not just the physical but also the psychological impacts of these medications. Future research should focus on elucidating the neuropsychological mechanisms of GLP-1RAs and exploring the potential risks and benefits in patients with preexisting mood disorders.

In conclusion, this case report contributes to the growing body of literature on the psychological side effects of GLP-1RAs, calling for a balanced approach to patient care that encompasses both physical and mental health considerations. As the use of these agents expands, clinicians should remain aware of the potential psychological impacts and ensure comprehensive monitoring and management strategies for their patients.

## Author contributions

**Conceptualization:** Yangliuqing He, Fenrong Liang.

**Data curation:** Yangliuqing He.

**Formal analysis:** Yangliuqing He.

**Funding acquisition:** Yangliuqing He.

**Investigation:** Yangliuqing He.

**Methodology:** Yangliuqing He.

**Project administration:** Yangliuqing He, Yuhan Wei.

**Resources:** Yangliuqing He.

**Software:** Yangliuqing He.

**Supervision:** Yangliuqing He, Yiming Wang.

**Validation:** Yangliuqing He.

**Visualization:** Yangliuqing He, Yiming Wang, Tianpei Ma.

**Writing – original draft:** Yangliuqing He.

**Writing – review & editing:** Yangliuqing He.
